# Cross-Cultural Adaptation and Validation of Incontinence Outcome Questionnaire for Serbian Population

**DOI:** 10.3390/medicina61030509

**Published:** 2025-03-16

**Authors:** Sladjana Kovacevic, Ivan Vukovic, Uros Bumbasirevic, Marko Zivkovic, Slavisa Savic, Zoran Bukumiric, Nikola Panajotovic, Petar Bulat, Bojan Cegar

**Affiliations:** 1 Department of Urology, General Hospital Pancevo, 26000 Pancevo, Serbia; nikolapanajotovic@hotmail.com; 2 Clinic of Urology, University Clinical Center of Serbia, 11000 Belgrade, Serbia; ivanvukovic.urolog@gmail.com (I.V.); urosbu@gmail.com (U.B.); markoziv91@gmail.com (M.Z.); bulat.petar@yahoo.com (P.B.); 3 Faculty of Medicine, University of Belgrade, 11000 Belgrade, Serbia; drsavics@yahoo.com; 4 Department of Urology, KBC Dr. Dragiša Mišovic, 11000 Belgrade, Serbia; 5 Institute of Medical Statistics and Informatics, Faculty of Medicine, University of Belgrade, 11000 Belgrade, Serbia; zoran.bukumiric@med.bg.ac.rs

**Keywords:** stress urinary incontinence, Incontinence Outcome Questionnaire, cross-cultural adaptation, validation studies, quality of life

## Abstract

*Background and Objectives*: Stress urinary incontinence (SUI) impacts 4–50% of adult women, frequently resulting in embarrassment, diminished self-esteem, and social withdrawal, significantly affecting quality of life. The aim of our study is to cross-culturally adapt and validate the Urinary Incontinence Outcome Questionnaire (IOQ) for the Serbian population and to assess the multifaceted impact of SUI on the quality of life among women. *Materials and Methods*: A cross-sectional study involved a total of 150 women: 100 undergoing surgical management for SUI (ST group) and 50 receiving non-surgical treatments, including vaginal estrogen, pessaries, electrical stimulation, or collagen fillers (NST group). The participants completed questionnaires on demographics, fatigue (MFI), anxiety and depression (HADS), and quality of life (SF-36), as well as the IOQ. *Results*: The ST group had a mean age of 60.0 ± 10.0 years, with 65% married, 65% with secondary education, 62% non-smokers, and 78% with comorbid conditions, primarily cardiovascular disease (59%). The NST group showed similar characteristics, with a significant difference in cardiovascular comorbidity (*p* = 0.049). All IOQ subscales demonstrated good internal consistency (Cronbach alpha > 0.7), except for the subscale Complications (Cronbach alpha = 0.440). The IOQ score for “Symptoms pre-operative” had the highest mean value (62.8 ± 18.6), while “Hospital Re-admission” had the lowest (303 ± 17.1). A comparison of the SF-36 scores showed significant differences in the Energy (*p* = 0.025) and Emotional well-being (*p* = 0.015) domains between the ST and NST groups. *Conclusions*: The Serbian version of the IOQ has been validated, demonstrating psychometric features that endorse its application in clinical and research contexts. This study highlights the significant impact of SUI on quality of life and the need for a comprehensive approach to treatment. The results emphasize the importance of addressing both the physical and psychological aspects of SUI to improve the lives of affected women.

## 1. Introduction

The International Continence Society (ICS) defines stress urinary incontinence (SUI) as the involuntary leakage of urine resulting from elevated abdominal pressure without detrusor contraction, occurring during activities like coughing, sneezing, or physical exertion [1]. SUI is estimated to impact between 4% and 50% of adult women, based upon the used definition of SUI, the time frame assessed, the symptom intensity, and the demographic examined. The prevalence increases with age, while obesity and smoking display consistent causal links with this health issue [2,3]. The extensive EPICONT study results indicate a progressive rise in the prevalence of moderate and severe urine incontinence with advancing age, alongside a notable increase in mild urinary incontinence during menopause [4]. Research has shown that among all forms of urine incontinence, the most significant rise in prevalence with age is noted in stress urinary incontinence (SUI) [5].

Management of SUI may be surgical or conservative, although nearly fifty percent of women with SUI do not pursue medical intervention [6,7]. Consequently, it is essential to educate this demographic group on the diverse array of therapy alternatives available. Non-surgical treatment methods for SUI include the use of pharmacotherapy, vaginal estrogen creams, gels, or patches, vaginal pessaries or cones, urethral inserts, electrical stimulation, external magnetic nerve stimulation of the pelvic floor muscles, and the application of collagen fillers in the urethra [8,9,10]. Surgical treatment for SUI involves miduretharal sling procedures with the use of polypropylene mesh, either via the transvaginal (TVT) or the transobturator (TOT) route; autologous slings; open or laparoscopic colposuspension; and bulking agents [11,12]. Acknowledging the various options available to patients, which differ in efficacy and safety profiles, a joint decision-making approach is essential when proposing any surgical therapies.

The presence of SUI is typically accompanied by feelings of embarrassment and reduced self-esteem, leading to the avoidance of social activities, which significantly disrupts the quality of life (QoL) for those affected [13]. Individuals with SUI often do not report having this condition, resulting in a diminished enjoyment of life. The most significant consequences of SUI include depression, anxiety, impaired sexual functioning, and reduced physical activity [14]. Although SUI adversely affects patients’ QOL, this influence is not invariably correlated with the intensity of the symptoms [15]. Additionally, research indicates that individuals with SUI can show symptoms of depression, while studies reveal that the incidence of anxiety among those with urinary incontinence is 50% higher than in the general population [16,17].

The management of SUI leads to enhanced quality of life, reduced anxiety, alleviated incontinence-related sexual dysfunction, and less discomfort [18,19]. However, the concept of success in SUI surgery can vary due to disparities between physician assessments and patient symptom perceptions [20]. Assessing the efficacy of interventions for SUI necessitates the use of validated instruments to gauge the results. The outcomes should be evaluated using objective metrics, including voiding diaries, stress tests, and pad tests, in addition to patient-reported questionnaires specific to SUI for the assessment of QoL and sexual function [21]. In 2007, Bjelic-Radisic et al. created and psychometrically validated the Incontinence Outcome Questionnaire (IOQ) to evaluate the quality of life with SUI, which may be utilized as a standalone assessment post-intervention, even in the absence of baseline pre-treatment data [22]. Furthermore, the IOQ is comprehensible and straightforward to respond to; the once-only post-treatment administration renders this validated instrument both practical and efficient. To achieve effectiveness in varied populations, these metrics must be culturally and linguistically tailored to accurately reflect the local patient experience. The aim of our study is to cross-culturally adapt and validate the IOQ for the Serbian population and to assess the multifaceted impact of SUI on the quality of life among women.

## 2. Materials and Methods

### 2.1. Study Design

A cross-sectional study was conducted from 1 June to 30 September 2024. Eligible patients following treatment for SUI were recruited at the Clinic of Urology, University Clinical Center of Serbia in Belgrade, and the Department of Urology, General Hospital in Pančevo, and completed the questionnaires in their respective institutions. The study was conducted in accordance with the institutional ethical board standards (Institutional Review Board of the General Hospital Pančevo, approval number 01-7814/23 (26 December 2023); the Ethics Committee of the Faculty of Medicine, University of Belgrade, Serbia, approval number 25/V-1 (22 May 2024); and the principles of the Declaration of Helsinki. All participants provided informed consent prior to their involvement in the study.

### 2.2. Selection of Participants

The study included 100 consecutive female patients who received surgical treatment for SUI in the surgical treatment (ST) group and 50 female patients with SUI who underwent non-surgical treatment (NST) during the same timeframe (March 2017 to June 2023), all of whom were regularly followed up with for a minimum of one year post-treatment. The dynamics of the hospital admissions for SUI treatment in these institutions determined the sample size. All eligible patients during the study period were included in the study. The inclusion criteria comprised patients over 18 years of age, having a confirmed diagnosis of SUI and having undergone either surgery or non-surgical treatment, and having provided consent to participate in the study. The exclusion criteria included the following: >80 years of age; a body mass index (BMI) > 37 kg/m^2^; the presence of a severe comorbidity (left ventricular ejection fraction (LVEF) ≤ 30%, NYHA IV, severe liver disease, aortic stenosis with ostium surface below 1 cm^2^, FEV1 on bronchodilatators below 50, VC below 60, hereditary coagulopathies without provision of preparations/lack of factors VII and VIII, myocardial infarction or stroke in the past six months); and the presence of major psychiatric conditions.

### 2.3. Procedures

The diagnosis of SUI was established following a thorough clinical history and a comprehensive physical examination, which included a standardized cough stress test conducted in either a supine or standing posture with 200–400 mL of fluid in the bladder.

The ST group comprised women that underwent a transvaginal synthetic midurethral sling insertion by the transobturator route. The procedure is performed in the lithotomy position following the placement of a 16 French urinary catheter. An incision is performed on the anterior vaginal wall 1 cm inferior to the external urethral meatus, measuring 2 cm in length. A sharp and blunt paraurethral dissection is carried out laterally and to the superior region of the ischiopubic ramus, and then the obturator membranes are pierced with the tips of the scissors. The introducer is thereafter advanced within the previously established dissection pathway until it penetrates the obturator membrane. Upon the extraction of both tubes through the skin incisions, the tape is subsequently positioned beneath the intersection of the mid and distal urethra, and the tension of the tape is tuned. The procedure lasts around 20 min, and the patient is discharged from the hospital either on the same day or the following day.

The NST group included female participants with SUI treated with vaginal estrogen creams, gels, or patches, the use of vaginal pessaries or rings, the use of urethral pads, electrical stimulation, external magnetic innervation of the muscles of the lower part of the pelvis, and the use of collagen fillers in the urethra.

### 2.4. Study Instruments

The questionnaire on demographic characteristics included questions related to age, marital status, place of residence, level of education, working status, lifestyle habits (smoking status, alcohol consumption, BMI), and data from personal history (benign and malignant diseases, chronic diseases, number of deliveries).

The Multidimensional Fatigue Inventory (MFI) [23] is a fatigue assessment scale consisting of 20 questions, divided into five domains: general fatigue, physical fatigue, reduced motivation, reduced activity, and mental fatigue. Each question is scored on a scale from 1 to 5. A higher total score denotes a higher degree of fatigue.

The Hospital Anxiety and Depression Scale (HADS) [24] is a valid and reliable scale for the self-assessment of anxiety and depression in hospitals and social environments. The HADS questionnaire consists of 14 questions (7 questions related to anxiety and 7 questions related to depression). The time required to fill out the questionnaire is from 2 to 5 min. The responses are scored on a scale of 0 to 3; the maximum score is 21 for anxiety and 21 for depression. A total score of 0–7 is considered normal, a score of 8–10 implies mild symptoms of anxiety/depression, a score of 11–15 moderate, and a score of 16–21 implies the presence of severe symptoms of anxiety/depression.

The SF-36 quality of life questionnaire [25] is a general questionnaire containing 36 questions covering eight domains: physical functioning, physical role limitations, emotional role limitations, social functioning, mental health, pain, vitality, and general health. The score for each individual domain ranges from 0 to 100, where a higher score indicates a better quality of life.

The Urinary Incontinence Outcome Questionnaire (IOQ) [22] is a valid and reliable instrument for assessing the quality of life after surgery for urinary incontinence. It can be used even if baseline or pre-operative data are not available. The questionnaire contains 27 questions, of which 4 refer to symptoms, 4 to complications, 12 to quality of life and satisfaction, 1 to problems with urinary incontinence, and 6 to demographic characteristics and the treatment itself. In this study, the validation and cultural adaptation of the IOQ in Serbian was carried out according to the internationally adopted practices. After obtaining permission from the original questionnaire’s author, two translators independently translated the questionnaire from German to Serbian, a process known as forward translation. Next, they created a harmonized Serbian version. The next step entailed a “backward translation” into German from Serbian by a third translator who had not seen the original version. A comparison was made between the original German version and the “back” translation, and a pre-final version of the questionnaire was created. This version was tested on a small number of respondents (pilot testing on 10 respondents) to ensure the clarity and understanding of the questions, and the necessary corrections were made. The final version of the questionnaire was then arrived at and validated on a sample of 100 respondents. During the validation process, the rate of missing answers and criterion validity (correlations with the scores of the general SF-36 questionnaire, which also measures quality of life) evaluated the acceptability of the questionnaire.

### 2.5. Statistical Analysis

Numerical data were presented as arithmetic means with standard deviation, while categorical data were presented by absolute frequencies with percentages. The comparison of different variables between the ST and NST groups was performed using the *t*-test for the numerical variables and the chi-square test for the categorical variables. The Cronbach alpha coefficient was used to test the internal consistency. The test–retest reliability was determined using the intraclass correlation coefficient, and values over 0.7 were considered satisfactory. The item-convergent validity was evaluated by calculating the correlation between each item and the scale it belongs to, while the discriminant validity was assessed by calculating the correlations of each item with the other scales. The criterion validity was assessed by the correlation coefficients between the IOQ and the SF-36 questionnaire, HADS, and MFI scores. All analyses were performed in SPSS (Statistical Package for Social Sciences), version 20.0. A *p*-value < 0.05 was considered statistically significant.

## 3. Results

Table 1 provides information on the demographic and clinical characteristics of the participants included in the study. The participants in the SIU surgical treatment group were on average 60.0 ± 10.0 years old. The majority of them were married (65.0%), had two children (65.0%), and had a secondary level of education (65.0%). In terms of habits, 62% of the participants in the SUI surgical treatment group were non-smokers, and 97% didn’t use alcohol (Table 1). More than three-quarters (78.0%) had some comorbid condition, most frequently CVD (59.0%). The participants in the SUI non-surgical treatment group were similar to those in the SIU surgical treatment group, with only one significant difference in the frequency of CVD comorbidity (*p* = 0.049) (Table 1).

Table 2 presents the descriptive data for all IOQ items. There were no missing values, favoring the maximum acceptability of the questionnaire. The highest mean value was observed for the item IOQ8—“Symptoms pre-operative” (62.8 ± 18.6)—and the lowest for the item IOQ 4—“Hospital Re-admission” (3.0 ± 17.1)—within the Complications subscale (Table 2).

All IOQ subscales demonstrated good internal consistency (Cronbach alpha >0.7) except for the subscale Complications (Cronbach alpha = 0.440). Additionally, the single IOQ items demonstrated good correlations with all IOQ subscale scores (Table 3). This pattern was also observed between different IOQ domains (Table 4). The results of the test–retest reliability analysis revealed that all scales demonstrated excellent intra-rater reliability, with intraclass correlation coefficients greater than 0.70 (Symptoms scale: 0.94; Complications scale: 0.96; Quality of life scale: 0.92; Satisfaction scale: 0.76; Quality of life/Satisfaction scale: 0.87; extended Quality of life/Satisfaction scale: 0.84).

Table 5 demonstrates the associations between the IOQ domains and HADS, MFI, and SF-36 total scores, as well as with the SF-36 domains and composite scores. The Symptoms subscale correlated significantly with the HADS total score (r = 0.209; *p* < 0.05), SF-36 Energy (r = −0.262, *p* < 0.01), SF-36 Emotional well-being (r = −0.221; *p* < 0.05), SF-36 Social functioning (r = −0.216, *p* < 0.05), SF-36 Mental health composite (r = −0.244; *p* < 0.05), and SF-36 Total score (r = −0.220; *p* < 0.05). The QOL subscale was associated with the HADS total score (r = 0.326; *p* < 0.01), SF-36 Emotional well-being (r = −0.220; *p* < 0.05), and SF-36 Social functioning (r = −0.208, *p* < 0.05). The Satisfaction subscale correlated with the SF-36 subscales Emotional well-being (r = −0.203; *p* < 0.05) and Social functioning (r = −0.198, *p* < 0.05). Finally, the subscale QOL/Satisfaction correlated with the HADS total score (r = 0.297; *p* < 0.01) and with the SF-36 subscales Emotional well-being (r = −0.235; *p* < 0.05) and Social functioning (r = −0.225, *p* < 0.05).

A comparison of the HADS, MFI, and SF-36 total and subscale scores between the ST and NST groups is presented in Table 6. The only significant differences were observed for the SF-36 Energy domain (62.3 ± 24.3 in the ST group and 53.3 ± 23.0 in the NST group, *p* = 0.025) and SF-36 Emotional well-being domain (75.1 ± 21.9 in the ST group and 67.9 ± 20.5 in the NST group, *p* = 0.015).

## 4. Discussion

The validation of the Serbian version of the Incontinence Outcome Questionnaire (IOQ) is a major step in improving the clinical evaluation of SUI in Serbian-speaking patients. Bjelic-Radisic et al. [22] previously stated the importance of employing validated questionnaires before and after treatment to evaluate the QoL alterations in women with UI. The deployment of a singular measurement tool, such as IOQ, tailored for evaluating the post-treatment QoL in women with SUI offers a pragmatic solution. This instrument, considered user-friendly, achieved high completion rates (96%) among a varied responder group, which indicates that streamlined assessment instruments may improve participation and yield significant insights into patient experiences following therapy.

The validation process followed internationally accepted standards for the translation and adaptation, including forward and backward translation, expert evaluation, and cognitive debriefing. These steps guaranteed that the Serbian rendition of the IOQ was both linguistically precise and culturally pertinent, essential for elucidating the intricacies of SUI’s effect on QoL across many cultural contexts. Furthermore, the criterion validity was assessed by correlating the IOQ subscales with established instruments such as the SF-36, HADS, and MFI. The Serbian IOQ demonstrated robust internal consistency across its subscales, with Cronbach alpha coefficients exceeding 0.7, signifying reliable measurement within each domain. Nevertheless, the Complications subscale exhibited a lower Cronbach alpha coefficient (0.440), indicating possible concerns regarding the item coherence or the inconsistency in the reporting of complications following treatment. This discovery requires additional examination, as indicated in comparable findings by the original questionnaire’s creator [22]. Cultural adaptation was essential, ensuring that the IOQ aligned with Serbian cultural values and linguistic practices. The cognitive debriefing refined the questionnaire to align with the local expressions and perceptions of incontinence, hence improving its relevance and acceptance among Serbian women. The cognitive interviews indicated slight terminological modifications required to align with colloquial language, therefore improving understanding among prospective respondents.

This research report further examines the complex effects of SUI on women’s QoL, highlighting notable disparities across different health domains as assessed by the SF-36 scale. The results demonstrate that women in the ST group exhibited higher scores in both the Energy (62.3 ± 24.3) and Emotional well-being (75.1 ± 21.9) domains relative to those in the NST group (Energy: 53.3 ± 23.0, *p* = 0.025; Emotional well-being: 67.9 ± 20.5, *p* = 0.015). The results underscore the significance of therapy type in affecting women’s QoL, consistent with prior work that stresses the value of personalized therapeutic strategies. The association between the Serbian IOQ and the SF-36 was notably important. The Symptoms subscale of the IOQ had a strong correlation with the SF-36 Energy domain (r = −0.262, *p* < 0.01) and the Emotional well-being domain (r = −0.221, *p* < 0.05), suggesting that incontinence symptoms adversely affect both dimensions of general well-being. The QoL subscale had substantial correlations with Emotional well-being (r = −0.220, *p* < 0.05) and Social functioning (r = −0.208, *p* < 0.05), highlighting the extensive QoL ramifications of SUI. The correlations indicate that the IOQ accurately reflects the multifaceted effects of SUI on daily functioning and emotional well-being, aligning with the results from extensive QoL research [14].

The literature review highlights the considerable prevalence of UI as a global health concern, with an estimated economic impact in the billions of US dollars. A comprehensive study and meta-analysis by Pizzol et al. [14] confirm that UI negatively impacts QoL; however, conventional treatments frequently do not produce significant benefits. The 2019 review and network meta-analysis further confirm that behavioral therapy is generally more efficient than pharmacological treatments in controlling both stress and urgency UI [26]. Nonetheless, the variable efficacy of behavioral interventions, especially in cases of mixed urine incontinence (MUI), presents issues for clinical practice and requires additional research. Additionally, two systematic studies without a formal meta-analysis supported these findings, indicating that women with UI report a diminished quality of life compared to their healthy counterparts [27]. Nevertheless, constraints like limited sample sizes impede the generalizability of these results. Multiple mechanisms may further elucidate the connection between UI and QoL. Individuals with UI frequently exhibit a greater incidence of comorbidities such as dementia and mobility impairments, which are acknowledged risk factors associated with diminished quality of life [28], particularly those selected for NST. Moreover, lifestyle factors such as hydration and diuretic consumption may further aggravate UI symptoms, highlighting the intricate relationship between physical health and QoL. These assertions can somewhat explain the disparities in QoL between the ST and NST groups in our study.

The impact of UI transcends simple inconvenience and the effect on QoL; it is associated with numerous adverse health consequences, such as sleep problems, urinary tract infections, falls, non-traumatic fractures, depression, and diminished work productivity [13,29,30]. Many older persons alarmingly view urinary incontinence as a normal part of aging, which may postpone their pursuit of therapy [31]. This misconception, coupled with the under-reporting of UI symptoms among women, perpetuates a loop of insufficient detection and treatment by healthcare providers. Research demonstrates that fewer than fifty percent of individuals impacted by urinary incontinence pursue assessment or treatment, highlighting the imperative for enhanced knowledge and education on this issue among patients and healthcare providers [7].

Moreover, our study assessed the relationship between SUI and psychological disturbances. The correlation between the IOQ and HADS was significant, particularly for the QoL subscale (r = 0.326, *p* < 0.01). This result not only confirms the IOQ’s capacity to assess the psychological impact of incontinence, but also corresponds with prior studies connecting SUI to elevated levels of anxiety and depression [17]. The Symptoms subscale demonstrated a strong association with the HADS total score (r = 0.209, *p* < 0.05), highlighting the mental health ramifications of incontinence. The link between the IOQ and the MFI was less pronounced; nonetheless, it still offered insight into the potential contribution of SUI to fatigue, as the Symptoms subscale correlated with the overall fatigue measures. This indicates that although fatigue may not be the central emphasis of the IOQ, it encompasses certain aspects of the potential physical depletion associated with incontinence treatment.

The psychological effects of UI are significant, as numerous women encounter reduced self-esteem, anxiety, depression, and social stigma [17,32]. The stigma surrounding urinary incontinence can result in considerable lifestyle changes, such as decreased physical activity and increased worry over public incontinence, which further aggravate mental health problems [33]. This phenomenon is especially evident among younger women who may be less prepared to manage the social ramifications of incontinence. Consequently, these insights necessitate a holistic approach to treatment that addresses not only the physical aspects of UI, but also the psychological and social dimensions.

It is essential to acknowledge the limitations inherent in the current study. The exclusive reliance on cross-sectional studies introduces the risk of reverse causality, where individuals with a pre-existing low QoL may experience UI, complicating the interpretation of the findings. The absence of matching between the participants with UI and the control groups raises concerns about potential biases that warrant consideration in future investigations. Lastly, while the test–retest reliability of the IOQ was not directly measured here, future studies should consider this aspect to assess the IOQ’s stability over time, particularly important for chronic conditions like SUI.

## 5. Conclusions

In conclusion, the complex relationship between UI and quality of life requires an extensive understanding of the condition’s wider consequences. The Serbian version of the IOQ has undergone validation, demonstrating psychometric features that support its application in clinical and research contexts. This validation method emphasizes the necessity of culturally responsive instruments in health measurement, guaranteeing that the evaluations are both precise and pertinent to the specific community. The IOQ now serves as a significant tool for assessing the treatment outcomes in Serbian women with SUI, facilitating enhanced patient care and additional research in this domain. A heightened focus on the physical and psychological aspects of UI, together with enhanced awareness and education, is crucial for improving the lives of individuals impacted by this condition.

## Figures and Tables

**Table 1:** Demographics and clinical profiles of participants.

Variable	ST Group (*n* = 100)	NST Group (*n* = 50)	*p*-Value
Age (years), mean ± SD	60.0 ± 10.0	62.1 ± 10.5	0.235
Marital status, *n* (%) Married/living with partner Single/divorced/widowed	68 (68.0) 32 (32.0)	34 (68.0) 16 (32.0)	1.000
Number of deliveries, *n* (%) Zero One Two Three Four	3 (3.0) 18 (18.0) 65 (65.0) 14 (14.0) 0 (0.0)	1 (2.0) 10 (20.0) 33 (66.0) 5 (10.0) 1 (2.0)	0.821
Number of children, *n* (%) Zero One Two Three Four	3 (3.0) 18 (18.0) 65 (65.0) 14 (14.0) 0 (0.0)	2 (4.0) 9 (18.0) 34 (68.0) 4 (8.0) 1 (2.0)	0.642
Place of residence, *n* (%) Rural Urban	43 (43.0) 57 (57.0)	22 (44.0) 28 (56.0)	0.907
Educational attainment, *n* (%) Primary Secondary College/University	19 (19.0) 65 (65.0) 16 (16.0)	12 (24.0) 28 (56.0) 10.0 (20.0)	0.912
Smoking status, *n* (%) Smoker Non-smoker	38 (38.0) 62 (62.0)	12 (24.0) 38 (76.0)	0.086
Number of cigarettes per day, mean ± SD	6.9 ± 10.2	3.5 ± 6.9	0.055
Alcohol consumption, *n* (%) Yes No	3 (3.0) 97 (97.0)	1 (2.0) 49 (98.0)	0.720
BMI (kg/m^2^), mean ± SD	28.8 ± 4.5	28.1 ± 4.3	0.385
Chronic diseases, *n* (%) No Yes	22 (22.0) 78 (78.0)	14 (28.6) 35 (71.4)	0.379
Malignant diseases, *n* (%) No Yes	99 (99.0) 1 (1.0)	49 (98.0) 1 (2.0)	0.615
Cardiovascular diseases, *n* (%) No Yes	41 (41.0) 59 (59.0)	29 (58.0) 21 (42.0)	0.049
Type 2 diabetes, *n* (%) No Yes	76 (76.0) 24 (24.0)	43 (86.0) 7 (14.0)	0.154
Locomotor, *n* (%) No Yes	85 (85.0) 15 (15.0)	47 (94.0) 3 (6.0)	0.110
Asthma, *n* (%) No Yes	91 (91.0) 9 (9.0)	47 (94.0) 3 (6.0)	0.523
Endocrine disease, *n* (%) No Yes	83 (83.0) 17 (17.0)	44 (88.0) 6 (12.0)	0.423

**Table 2:** Descriptive statistics for IOQ items.

Item	Question	Number	Mean	SD	Median
Subscale: Symptoms					
IOQ1	Pain	100	23.6	27.8	20
IOQ9	Symptoms post-operative	100	10.0	23.8	0
IOQ20	OAB pre-operative	100	16.0	36.8	0
IOQ21	Change in OAB symptoms pre- or post-operative	100	5.8	15.4	0
Subscale: Complications					
IOQ2	Urinary infection	100	24.0	42.9	0
IOQ3	Other infection	100	23.0	42.3	0
IOQ4	Hospital Re-admission	100	3.0	17.1	0
IOQ19	Residual urine	100	8.3	15.9	0
Subscale: Quality of life					
IOQ5	Felt tired/drained/lacking	100	20.5	30.2	0
IOQ6	Felt irritable/snappy	100	40.0	26.6	50
IOQ7	Felt depressed/tearful	100	20.0	27.5	0
IOQ10	Global evaluation of health	100	35.0	21.0	25
IOQ12	Limitations in daily activities	100	22.0	26.9	0
IOQ13	Change in sex life	100	31.7	27.4	33.3
IOQ14	Change in feeling about body	100	12.0	22.6	0
Subscale: Satisfaction					
IOQ11	Symptom changes pre- and post-operative	100	9.5	23.0	0
IOQ15	Time of recovery	100	33.7	31.6	33.3
IOQ16	Satisfaction with information	100	35.5	26.9	50
IOQ17	Improvement in well-being	100	12.5	18.3	0
IOQ18	Recommending operation	100	5.3	17.5	0
Single item					
IOQ8	Symptoms pre-operative	100	62.8	18.6	75

**Table 3:** Internal consistency of IOQ questionnaire.

	Symptoms	Complications	QOL	Satisfaction	QOL/Satisf.
Subscale: Symptoms (α = 0.712)					
IOQ1	0.491 **	0.212 *	0.118	0.189	0.164
IOQ9	0.846 **	0.291 **	0.454 **	0.509 **	0.528 **
IOQ20	0.879 **	0.162	0.199 *	0.251 *	0.244 *
IOQ21	0.852 **	0.197 *	0.318 **	0.397 **	0.389 **
Subscale: Complications (α = 0.440)					
IOQ2	0.239 *	0.798 **	0.183	0.188	0.205 *
IOQ3	0.032	0.789 **	0.057	0.065	0.067
IOQ4	0.140	0.392 **	0.046	−0.016	0.022
IOQ19	0.487 **	0.292 **	0.484 **	0.586 **	0.583 **
Subscale: Quality of life (α = 0.744)					
IOQ5	0.220 *	0.119	0.794 **	0.456 **	0.720 **
IOQ6	0.118	0.154	0.505 **	0.264 **	0.446 **
IOQ7	0.144	0.179	0.751 **	0.402	0.667 **
IOQ10	0.275 **	0.246 *	0.570 **	0.532 **	0.613 **
IOQ12	0.248 *	0.134	0.668 **	0.411 **	0.619 **
IOQ13	0.090	−0.075	0.395 **	0.216 *	0.353 **
IOQ14	0.421 **	0.354 **	0.723 **	0.530 **	0.709 **
Subscale: Satisfaction (α = 0.743)					
IOQ11	0.336 *	0.172	0.620 **	0.793 **	0.767 **
IOQ15	0.240 *	0.144	0.461 **	0.748 **	0.645 **
IOQ16	0.118	0.158	0.218 *	0.642 **	0.440 **
IOQ17	0.496 **	0.181	0.560 **	0.752 **	0.710 **
IOQ18	0.417 **	0.296 **	0.444 **	0.657 **	0.591 **
Subscale: Quality of life/Satisfaction (α = 0.833)					
IOQ5	0.220 *	0.119	0.794 **	0.456 **	0.720 **
IOQ6	0.118	0.154	0.505 **	0.264 **	0.446 **
IOQ7	0.144	0.179	0.751 **	0.402 **	0.667 **
IOQ10	0.275 **	0.246 *	0.570 **	0.532 **	0.613 **
IOQ12	0.248 *	0.134	0.668 **	0.411 **	0.619 **
IOQ13	0.090	−0.075	0.395 **	0.216 *	0.353 **
IOQ14	0.421 **	0.354 **	0.723 **	0.530 **	0.709 **
IOQ11	0.336 **	0.172	0.620 **	0.793 **	0.767 **
IOQ15	0.240 *	0.144	0.461 **	0.748 **	0.645 **
IOQ16	0.118	0.158	0.218 *	0.642 **	0.440 **
IOQ17	0.496 **	0.181	0.560 **	0.752 **	0.710 **
IOQ18	0.417 **	0.296 **	0.444 **	0.657 **	0.591 **
Symptoms pre-operative					
IOQ8	−0.139	−0.004	−0.060	−0.001	−0.038

* *p* < 0.05; ** *p* < 0.01.

**Table 4:** Correlations between IOQ domains.

	Subscale: Symptoms	Subscale: Complications	Subscale: QOL	Subscale: Satisfaction	Subscale: QOL/ Satisfaction
Subscale: Symptoms	/				
Subscale: Complications	0.275 **	/			
Subscale: Quality of life	0.332 **	0.237 *	/		
Subscale: Satisfaction	0.413 **	0.252 *	0.625 **	/	
Subscale: QOL/ Satisfaction	0.405 **	0.269 **	0.931 **	0.867	/

* *p* < 0.05; ** *p* < 0.01.

**Table 5:** Correlation between IOQ domains and HADS, MFI, and SF-36 scores.

Score	Subscale: Symptoms	Subscale: Complications	Subscale: QOL	Subscale: Satisfaction	Subscale: QOL/ Satisfaction
HADS total score	0.209 *	0.018	0.326 **	0.189	0.297 **
MFI total score	0.032	−0.070	−0.117	−0.010	−0.079
SF-36 Physical functioning	−0.013	0.080	−0.053	−0.026	−0.046
SF-36 Role limitations due to physical health	−0.135	0.033	−0.073	−0.055	−0.073
SF-36 Role limitations due to emotional problems	−0.161	0.009	−0.042	−0.091	−0.070
SF-36 Energy	−0.262 **	−0.018	−0.157	−0.196	−0.192
SF-36 Emotional well-being	−0.221 *	−0.096	−0.220 *	−0.203 *	−0.235 *
SF-36 Social functioning	−0.216 *	−0.021	−0.208 *	−0.198 *	−0.225 *
SF-36 Pain	−0.184	−0.075	−0.160	−0.173	−0.183
SF-36 General health	−0.185	−0.096	−0.074	−0.138	−0.111
SF-36 Physical health composite	−0.161	−0.011	−0.113	−0.118	−0.128
SF-36 Mental health composite	−0.244 *	−0.029	−0.167	−0.191	−0.196
SF-36 Total score	−0.220 *	−0.022	−0.152	−0.167	−0.175

* *p* < 0.05; ** *p* < 0.01.

**Table 6:** Comparison of HADS, MFI, and SF-36 scores between ST and NST groups.

Score	ST Group	NST Group	*p*-Value
HADS total score, median (range)	11 (0–32)	14 (3–32)	0.129
MFI total score, mean ± SD	58.5 ± 6.8	57.4 ± 7.6	0.365
SF-36 Physical functioning	63.8 ± 24.2	59.6 ± 28.2	0.451
SF-36 Role limitations due to physical health	44.8 ± 34.0	54.0 ± 35.8	0.128
SF-36 Role limitations due to emotional problems	63.3 ± 38.6	64.0 ± 39.2	0.916
SF-36 Energy	62.3 ± 24.3	53.3 ± 23.0	0.025 *
SF-36 Emotional well-being	75.1 ± 21.9	67.9 ± 20.5	0.015 *
Sf-36 Social functioning	68.4 ± 27.7	68.5 ± 22.9	0.802
SF-36 Pain	61.6 ± 27.4	54.2 ± 25.5	0.091
SF-36 General health	57.8 ± 20.5	51.2 ± 21.2	0.066
SF-36 Physical health composite	57.0 ± 21.3	54.7 ± 22.7	0.537
SF-36 Mental health composite	67.3 ± 23.9	63.4 ± 20.2	0.196
SF-36 Total score	62.1 ± 21.1	59.1 ± 20.1	0.308

* *p* < 0.05.

## Data Availability

The data supporting the reported results can be found upon request in the form of datasets available at the Department of Urology, General Hospital in Pančevo, and the Clinic of Urology, University Clinical Centre of Serbia.
